# Mental disorder symptoms during the COVID-19 pandemic in Latin America – a systematic review and meta-analysis

**DOI:** 10.1017/S2045796021000767

**Published:** 2022-04-19

**Authors:** Stephen X. Zhang, Kavita Batra, Wen Xu, Tao Liu, Rebecca Kechen Dong, Allen Yin, Andrew Yilong Delios, Bryan Z. Chen, Richard Z. Chen, Saylor Miller, Xue Wan, Wenping Ye, Jiyao Chen

**Affiliations:** 1 Faculty of Professions, University of Adelaide, Adelaide, SA 5005, Australia; 2 Office of Research, School of Medicine, University of Nevada, Las Vegas, NV 89102, USA; 3 Nottingham University Business School China, University of Nottingham Ningbo China, Ningbo 315100, China; 4 College of Economics and Management, Southwest University, Chongqing 400716, China; 5 Business School, University of South Australia, Adelaide, SA 5001, Australia; 6 School of Humanities, Southeast University, Nanjing 211189, China; 7 Department of Psychology, University of Adelaide, Adelaide, SA 5001, Australia; 8 Crescent Valley High School, Corvallis, OR 97330, USA; 9 Crescent Valley High School, Corvallis, OR 97330, USA; 10 College of Business, Oregon State University, Oregon, OR 97331, USA; 11 School of Economics and Management, Tongji University, Shanghai 200092, China; 12 School of Management, Jinan University, Guangzhou 510632, China; 13 College of Business, Oregon State University, Corvallis, OR 97331, USA

**Keywords:** COVID-19, healthcare workers, Latin America, mental health, meta-analysis, prevalence

## Abstract

**Aims:**

There is a lack of evidence related to the prevalence of mental health symptoms as well as their heterogeneities during the coronavirus disease 2019 (COVID-19) pandemic in Latin America, a large area spanning the equator. The current study aims to provide meta-analytical evidence on mental health symptoms during COVID-19 among frontline healthcare workers, general healthcare workers, the general population and university students in Latin America.

**Methods:**

Bibliographical databases, such as *PubMed, Embase, Web of Science, PsycINFO* and *medRxiv*, were systematically searched to identify pertinent studies up to August 13, 2021. Two coders performed the screening using predefined eligibility criteria. Studies were assigned quality scores using the Mixed Methods Appraisal Tool. The double data extraction method was used to minimise data entry errors.

**Results:**

A total of 62 studies with 196 950 participants in Latin America were identified. The pooled prevalence of anxiety, depression, distress and insomnia was 35%, 35%, 32% and 35%, respectively. There was a higher prevalence of mental health symptoms in South America compared to Central America (36% *v*. 28%, *p* < 0.001), in countries speaking Portuguese (40%) *v.* Spanish (30%). The pooled prevalence of mental health symptoms in the general population, general healthcare workers, frontline healthcare workers and students in Latin America was 37%, 34%, 33% and 45%, respectively.

**Conclusions:**

The high yet heterogenous level of prevalence of mental health symptoms emphasises the need for appropriate identification of psychological interventions in Latin America.

## Introduction

Latin America, consisting of 33 countries or territories, has had the second-highest amount of coronavirus disease 2019 (COVID-19) cases and deaths per capita (Burki, 2020; World Health Organization, 2020; Ríos, 2021). Latin America is vulnerable to the destructive outbreak for several reasons including long-standing structural and socioeconomic inequities (Carvalho *et al*., 2015; Dávila-Cervantes and Agudelo-Botero, 2019; Burki, 2020) over 20% of the population in poverty, lack of healthcare access, underfunded healthcare systems, poor governance or political dynamics, a high burden of chronic and metabolic health conditions and lack of preparedness to fight the pandemic (Malta *et al*., 2020). Reportedly, there is a considerable increase in psychological morbidities among several demographic groups, including healthcare workers, the general population and students (Campos *et al*., 2021*b*). Latin America is a vast area where tropical regions span across almost all countries and regional disparities on mental health have been reported (Malta *et al*., 2020), but we still lack evidence on the prevalence of mental health symptoms as well as their heterogeneities during the COVID-19 pandemic.

Recently, meta-analyses have provided early global evidence on the prevalence of mental health symptoms across groups, including healthcare workers, the general population and students (Batra *et al*., 2020; Luo *et al*., 2020; Pappa *et al*., 2020). These reports included very few studies based on Latin American samples. With emerging studies on mental health in Latin America, it is critical to synthesise meta-analytical evidence to provide integrated data on mental health among key demographic groups in Latin America during the COVID-19 pandemic. Therefore, this meta-analysis aims to investigate the pooled prevalence of mental health symptoms during the COVID-19 pandemic among frontline healthcare workers, general healthcare workers, the general population and university students in Latin America. We first perform subgroup analysis for Latin America based on South America (a majority but not all countries are in tropical regions) and Central America (all countries are entirely tropical).

## Methods

### Protocol registration

We followed the Preferred Reporting Items for Systematic Reviews and Meta-Analyses (PRISMA) statement 2020 (Liberati *et al*., 2009) to guide our meta-analysis and registered it with the International Prospective Register of Systematic Reviews (PROSPERO: CRD42020224458).

### Eligibility criteria

The search targeted observational studies that assessed the prevalence of psycho-morbid symptoms of anxiety, depression, distress and insomnia among frontline healthcare workers, general healthcare workers, the general population aged 18 years or above and university students in Latin America. A priori inclusion criteria were established to identify eligible studies that used established psychometric survey tools, used the English language, and were available as full-texts. Studies that targeted other populations, including children, adolescents and certain subgroups (e.g., pregnant women), were excluded. Other study designs, such as reviews and meta-analyses, qualitative, mixed methods, case reports, studies published only as abstracts, biochemical and experimental studies, or articles lacking the use of robust psychometric instruments or with an ambiguous methodology to identify prevalence were also excluded. Studies based on non-Latin American countries were excluded. Studies with unclear methodology and results were reviewed carefully, and a researcher (WX) attempted to contact authors to seek the information in several instances: (1) if the study reported estimates for both targeted and excluded populations, posing challenges for us to delineate the prevalence rate for the population of interest to our study; (2) if the study did not report the prevalence as proportions; (3) if the study did not specify cut-off scores for levels of severity; or (4) if the study was missing crucial information such as response rate, duration of data collection and gender distribution.

### Data sources and search strategy

This meta-analysis is part of a large project on meta-analysis of mental health symptoms during COVID-19. Bibliographic databases, such as *PubMed*, *Embase*, *PsycINFO* and *Web of Science*, were searched on 13 August 2021. *medRxiv* was also searched for preprints. Search algorithms specific to each database were used to yield a comprehensive pool of literature. A detailed search strategy appears in online Supplementary Table S1.

### Phases of screening

A researcher (JC) exported the search results from various databases into Endnote to remove duplicates and then imported them into Rayyan for subsequent screening. Two reviewers (AD & BZC) independently screened the titles and abstracts of all papers in accordance with the prespecified eligibility criteria. The eligible abstracts proceeded to full-text screening for possible inclusion. Any conflicts between reviewers were resolved by a third reviewer (RKD).

### Data extraction

A codebook was developed for standardisation and consistency. The final studies included from the screening process were sent to three groups (two reviewers in each group, WX & AY, BZC & AD, RZC & SM) for thorough investigation and extraction of relevant data elements into a coding book. Standardised codes were used to record pertinent variables, including author, title, country, duration of data collection, study design, population, sample size, response rate, female proportion, mean age, psychological outcome, severity level of outcome, type of survey instruments with cut-off scores and prevalence of psycho-morbid events. The severity of psychological outcomes of interest was coded as above mild, moderate above and severe levels (if available). The studies that reported only mild, moderate, and severe prevalence data were recoded into mild above, moderate above and severe prevalence for consistency purposes. The severity levels in studies that only reported the overall prevalence were determined based on cut-off scores (if available). After finishing independent coding, all the extracted data elements were subject to a second round of review by the coders to identify any discrepancies. In case of disagreements, a third reviewer (WX or TL) helped to achieve consensus through re-verification and discussion.

### Risk of bias (RoB) assessment

The Mixed Methods Appraisal Tool (MMAT) with seven questions was used as a quality assessment tool (Hong *et al*., 2018; Pablo *et al*., 2020; Usher *et al*., 2020). Two reviewers independently assessed and assigned scores to the studies using the tool dictionary and guidelines. Disagreements were resolved through discussion with the lead reviewer (RKD). The quality scores ranged from 0 to 7 (highest quality). Studies were categorised as high, medium, or low quality if they attained the score of 6, 5 to 6, or <5, respectively.

### Effect measure and data analysis

Using Version 16.1 of Stata (metaprop package), a random-effects model was used to compute the pooled estimates of outcome prevalence between populations by assuming that these studies are randomly selected from their targeted populations in Latin America to generalise our results to comparable studies in the region (Borenstein *et al*., 2021). We computed prediction intervals to show the range of the effect sizes across studies (Borenstein *et al*., 2017). The *I*^2^ statistic was used to calculate variance difference from effect sizes in order to quantify heterogeneity rather than sampling error (Higgins *et al*., 2019). Visual inspection of the Doi plot and the Luis Furuya–Kanamori (Furuya-Kanamori *et al*., 2018) index were used to assess publication bias (Kounou *et al*., 2020; Yitayih *et al*., 2020). The event ratio was used as the primary effect measure for the pooled estimates.

## Results

### Screening of studies

**Fig. 1.**
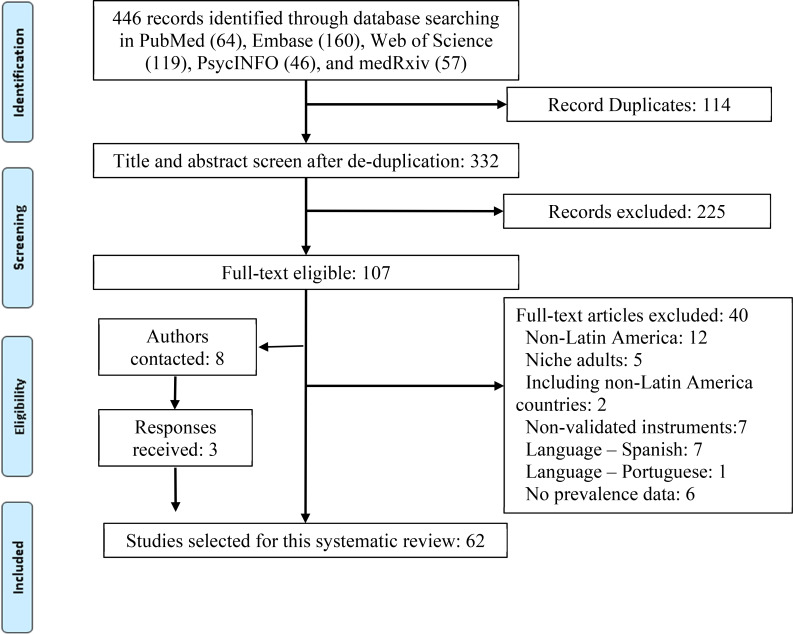
PRISMA flow diagram.

A total of 446 records were identified through searching bibliographical databases and other sources (Fig. 1). After removing 114 duplicates, a total of 332 records advanced to the screening phase. After excluding 225 records that did not pass the title and abstract screening, 107 records were identified as eligible for full-text screening. Among them, 40 papers were excluded for different reasons. For example, we excluded seven papers in Spanish and one paper in Portuguese. We sent emails to the authors of eight studies, to request missing critical information; three studies provided new prevalence data and were included in the final pool. Therefore, 62 studies, focused on populations in Latin America, were used in the final data extraction and analysis (online Supplementary Table S2).

### Study characteristics

A total of 65 unique samples from 62 studies involving 196 950 participants from Latin America were included in this meta-analysis (Badellino *et al*., 2020, 2022; Campos *et al*., 2020, 2021*a*, 2021*b*; Chen *et al*., 2021*a*, 2021*b*; Civantos *et al*., 2020; Cortés-Álvarez *et al*., 2020; Dal'Bosco *et al*., 2020; De Boni *et al*., 2020; Fernández *et al*., 2020; Giardino *et al*., 2020; Guiroy *et al*., 2020; Malgor *et al*., 2020; Martinez *et al*., 2020; Medeiros *et al*., 2020; Mier-Bolio *et al*., 2020; Monterrosa-Castro *et al*., 2020; Mora-Magaña *et al*., 2020; Passos *et al*., 2020; Paz *et al*., 2020; Samaniego *et al*., 2020; Schuch *et al*., 2020; Yáñez *et al*., 2020; Antiporta *et al*., 2021; Boluarte-Carbajal *et al*., 2021; Brito-Marques *et al*., 2021; Cayo-Rojas *et al*., 2021; Cénat *et al*., 2021; Dantas *et al*., 2021; de Oliveira Andrade *et al*., 2021; Espinosa-Guerra *et al*., 2021; Esteves *et al*., 2021; Fernandez *et al*., 2021; Ferreira *et al*., 2021; Feter *et al*., 2021; Flores-Torres *et al*., 2021; García-Espinosa *et al*., 2021; Goularte *et al*., 2021; Landaeta-Díaz *et al*., 2021; Loret de Mola *et al*., 2021; Mautong *et al*., 2021; Mendonca *et al*., 2021; Mota *et al*., 2021; Nayak *et al*., 2021; Puccinelli *et al*., 2021; Ribeiro *et al*., 2021; Schmitt Jr *et al*., 2021; Scotta *et al*., 2021; Serafim *et al*., 2021; Souza *et al*., 2021; Torrente *et al*., 2021*a*, 2021*b*; Villela *et al*., 2021; Vitorino *et al*., 2021; Werneck *et al*., 2021; Zhang *et al*., 2021*a*, 2021*c*; da Silva Júnior *et al*., 2021; Robles *et al*., 2021) (Table 1 and online Supplementary Table S2). Some studies include multiple independent samples. For example, one study examined the prevalence of both general healthcare workers and frontline healthcare workers. Among them, 35 samples (53.85%) were of general populations (Passos *et al*., 2020; Antiporta *et al*., 2021; Boluarte-Carbajal *et al*., 2021; de Oliveira Andrade *et al*., 2021; Espinosa-Guerra *et al*., 2021; Ferreira *et al*., 2021; Landaeta-Díaz *et al*., 2021; Mautong *et al*., 2021; Ribeiro *et al*., 2021; Schmitt Jr *et al*., 2021; Souza *et al*., 2021; Torrente *et al*., 2021*b*; Vitorino *et al*., 2021; Badellino *et al*., 2022), two samples (3.08%) were of frontline healthcare workers (Dal'Bosco *et al*., 2020; Robles *et al*., 2021), 19 samples (29.22%) were from general healthcare workers (Chen *et al*., 2020; Civantos *et al*., 2020; Giardino *et al*., 2020; Guiroy *et al*., 2020; Malgor *et al*., 2020; Monterrosa-Castro *et al*., 2020; Mora-Magaña *et al*., 2020; Samaniego *et al*., 2020; Yáñez *et al*., 2020; Zhang *et al*., 2021*a*; Villela *et al*., 2021; Nayak *et al*., 2021; Dantas *et al*., 2021; Mota *et al*., 2021; Brito-Marques *et al*., 2021; Campos *et al*., 2021*b*; Mier-Bolio *et al*., 2020; Robles *et al*., 2021) and nine samples (13.85%) were based on university students (Medeiros *et al*., 2020; Campos *et al*., 2021*a*; Cayo-Rojas *et al*., 2021; Esteves *et al*., 2021; Fernandez *et al*., 2021; García-Espinosa *et al*., 2021; Mendonca *et al*., 2021; Scotta *et al*., 2021; da Silva Júnior *et al*., 2021). Of the 62 studies, 32 were from Brazil (49.22%) (Table 1). Except for three (4.84%) longitudinal cohort studies (Feter *et al*., 2021; Flores-Torres *et al*., 2021; Loret de Mola *et al*., 2021), the majority of the studies were cross-sectional (95.16%). The sample size varied from 62 to 196 950 participants. The participation rates varied from 11.4% to 100.0% with a median value of 72.25%. The female proportions among the 65 samples varied from 3.4% to 89.8% with a median of 72.25%.

**Table 1.** Characteristics of the studies on mental health in Latin America during the COVID-19 pandemic

Characteristics	Total number of studies/samples^a^	Percent	Level of analysis
Overall	62/65	100	
Outcome^b^		-	Prevalence
Anxiety	95	42.79	
Depression	87	39.19	
Distress	21	9.46	
Insomnia	19	8.56	
Severity^b^			Prevalence
Above mild	77	34.68	
Above moderate	87	39.19	
Above severe	52	23.42	
Overall	6	2.71	
Population			Sample
Frontline HCWs	2	3.08	
General HCWs	19	29.22	
General population	35	53.85	
Students	9	13.85	
Sampling country			Sample
Argentina	8	12.31	
Bolivia	1	1.54	
Brazil	32	49.22	
Chile	1	1.54	
Colombia	1	1.54	
Ecuador	3	4.62	
Haiti	1	1.54	
Mexico	8	12.30	
Panama	1	1.54	
Paraguay	1	1.54	
Peru	6	9.23	
Trinidad and Tobago	1	1.54	
Mixed	1	1.54	
Quality			Study
High	30	48.39	
Medium	32	51.61	
*Design*			Study
Cohort	3	4.84	
Cross-sectional	59	95.16	
Publication			Study
Preprint	4	6.45	
Published	58	93.55	
Overall	Mean (median)	Range	
Number of participants	3030 (671)	31–57 446	Sample
Female proportion	67.9% (72.25%)	3.4–89.8%	Sample
Response rate	66.0% (73.7%)	11.4–100%	Sample

a Some studies include multiple independent samples. For example, one study^47^examined the prevalence of both general healthcare workers and frontline healthcare workers.

b One independent sample in a study may report anxiety, depression and insomnia at the levels of mild above, moderate above and severe. Therefore, the total number of prevalence is larger than the total number of independent samples.

### Estimates of pooled prevalence of psychological morbidity symptoms

**Fig. 2.**
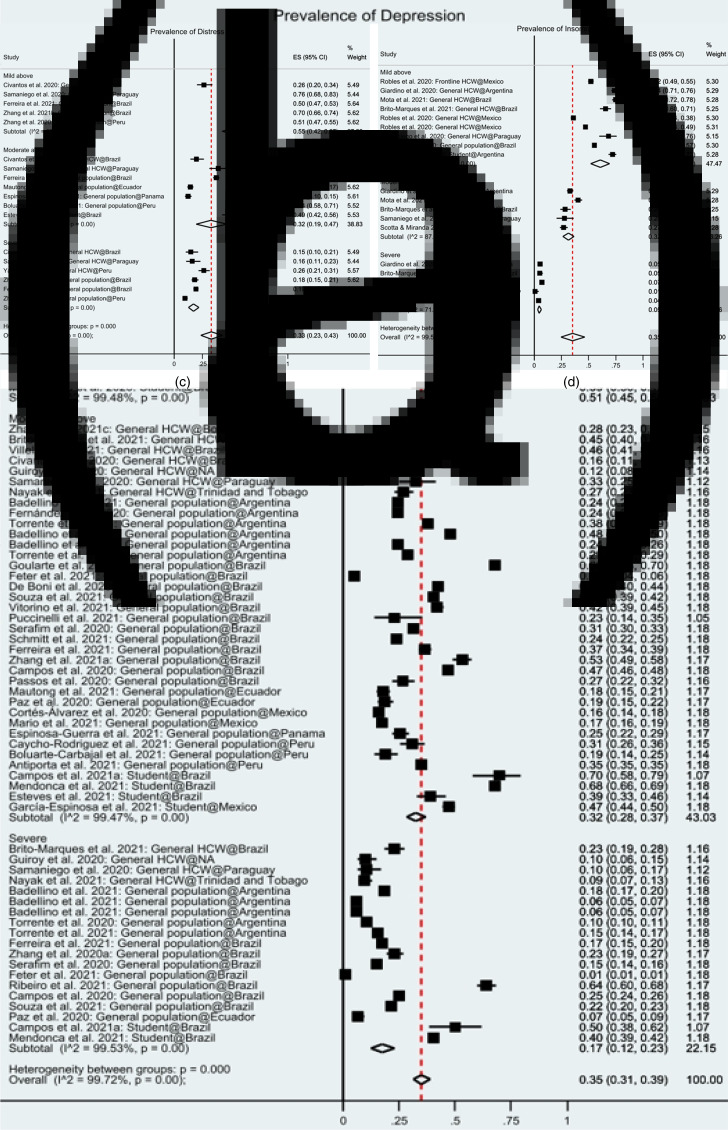
The square markers indicate the prevalence of insomnia symptoms among population groups of interest. The diamonds represent the pooled estimates. (a) Forest plot indicating the pooled prevalence of anxiety among included studies. (b) Forest plot indicating the pooled prevalence of depression among included studies. (c) Forest plot indicating the pooled prevalence of distress among included studies. (d) Forest plot indicating the pooled prevalence of insomnia among included studies.

In Latin America, 56 samples from 54 studies reported the prevalence of anxiety symptoms among 128 060 participants (Badellino *et al*., 2020; Campos *et al*., 2020, 2021*a*, 2021*b*; Chen *et al*., 2020; Civantos *et al*., 2020; Cortés-Álvarez *et al*., 2020; Dal'Bosco *et al*., 2020; De Boni *et al*., 2020; Fernández *et al*., 2020; Malgor *et al*., 2020; Martinez *et al*., 2020; Medeiros *et al*., 2020; Mier-Bolio *et al*., 2020; Monterrosa-Castro *et al*., 2020; Mora-Magaña *et al*., 2020; Passos *et al*., 2020; Paz *et al*., 2020; Samaniego *et al*., 2020; Schuch *et al*., 2020; Yáñez *et al*., 2020; Boluarte-Carbajal *et al*., 2021; Cayo-Rojas *et al*., 2021; Cénat *et al*., 2021; Dantas *et al*., 2021; de Oliveira Andrade *et al*., 2021; Espinosa-Guerra *et al*., 2021; Fernandez *et al*., 2021; Ferreira *et al*., 2021; Feter *et al*., 2021; Flores-Torres *et al*., 2021; García-Espinosa *et al*., 2021; Giardino *et al*., 2020; Goularte *et al*., 2021; Landaeta-Díaz *et al*., 2021; Loret de Mola *et al*., 2021; Mautong *et al*., 2021; Mendonca *et al*., 2021; Nayak *et al*., 2021; Puccinelli *et al*., 2021; Ribeiro *et al*., 2021; Serafim *et al*., 2021; Souza *et al*., 2021; Torrente *et al*., 2021*a*, 2021*b*; Vitorino *et al*., 2021; Werneck *et al*., 2021; Zhang *et al*., 2021*a*, 2021*b*; Caycho-Rodriguez *et al*., 2022;; da Silva Júnior *et al*., 2021; Robles *et al*., 2021). Among all the anxiety survey tools used, the Generalised Anxiety Symptoms 7-items scale (GAD-7) was the most common (51.85%), followed by the Depression, Anxiety and Stress Scale – 21 Items (DASS-21) (18.52%), the Hospital Anxiety and Depression Scale (HADS) (9.26%), Beck Anxiety Inventory (BAI) (3.70%) and nine others (each 1.85%). The cut-off values to determine the overall prevalence as well as severe anxiety varied across studies. In the random-effects model, the pooled prevalence of anxiety was 35% (95% CI: 31–38%) in the 54 studies (Fig. 2a). This finding suggests that, on average, 35% of the adults in Latin America had anxiety symptoms during COVID-19. Based on a normal distribution, its prediction internal is 5−75%, and the prevalence of anxiety symptoms in any comparable study will fall in this range.

A total of 49 samples from 46 studies reported the prevalence of depression among 139 559 respondents (Badellino *et al*., 2020, 2022; Campos *et al*., 2020; Civantos *et al*., 2020; Cortés-Álvarez *et al*., 2020; Dal'Bosco *et al*., 2020; De Boni *et al*., 2020; Fernández *et al*., 2020; Giardino *et al*., 2020; Guiroy *et al*., 2020; Martinez *et al*., 2020; Medeiros *et al*., 2020; Mora-Magaña *et al*., 2020; Passos *et al*., 2020; Paz *et al*., 2020; Samaniego *et al*., 2020; Schuch *et al*., 2020; Antiporta *et al*., 2021; Boluarte-Carbajal *et al*., 2021; de Oliveira Andrade *et al*., 2021; Espinosa-Guerra *et al*., 2021; Ferreira *et al*., 2021; Feter *et al*., 2021; García-Espinosa *et al*., 2021; Goularte *et al*., 2021; Loret de Mola *et al*., 2021; Mautong *et al*., 2021; Mendonca *et al*., 2021; Nayak *et al*., 2021; Puccinelli *et al*., 2021; Ribeiro *et al*., 2021; Schmitt Jr *et al*., 2021; Serafim *et al*., 2021; Souza *et al*., 2021; Torrente *et al*., 2021*a*, 2021*b*; Villela *et al*., 2021; Zhang *et al*., 2021*a*, 2021*b*; Caycho-Rodriguez *et al*., 2022; Robles *et al*., 2021). Among all the depression survey tools, the Patient Health Questionnaire (PHQ)-9 was the most frequently used (50%), followed by DASS-21 (21.74), HADS (10.87%), the Centre for Epidemiologic Studies Depression Scale (CESD) (4.35%) and six others (each 2.17%). Analysing the random-effects model, the pooled prevalence of depression was 35% (95% CI: 31−39%) among the 46 studies (Fig. 2b). This finding suggests that, on average, 35% of the adults in Latin America had depression symptoms during COVID-19. Its prediction internal is 7−71%.

Thirteen studies studied mental distress among 10 335 participants (Chen *et al*., 2020; Civantos *et al*., 2020; Cortés-Álvarez *et al*., 2020; Fernández *et al*., 2020; Reidy, 2020; Samaniego *et al*., 2020; Yáñez *et al*., 2020; Boluarte-Carbajal *et al*., 2021; Espinosa-Guerra *et al*., 2021; Ferreira *et al*., 2021; Zhang *et al*., 2021*c*). Among all the distress survey tools, DASS-21 was the most frequently used (30.77%), followed by COVID-19 Peritraumatic Distress Index (CPDI), Impact of Event Scale – Revised (IES) and K6 (15.38% each) and three others (7.69% each). In the random-effects model, the pooled prevalence of distress was 32% (95% CI: 25–40%) (Fig. 2c). This finding suggests that, on average, 32% of the adults in Latin America had distress symptoms during COVID-19. Its prediction interval is 1–79%.

Nine samples from seven studies (Giardino *et al*., 2020; Samaniego *et al*., 2020; Brito-Marques *et al*., 2021; Goularte *et al*., 2021; Mota *et al*., 2021; Scotta *et al*., 2021; Robles *et al*., 2021) studied insomnia among 12 134 respondents. The Insomnia Severity Index (ISI) (71.43%) was used most often, followed by Diagnostic and Statistical Manual (DSM) (28.57). In the random-effects model, the pooled prevalence of insomnia was 35% (95% CI: 25–46%) (Fig. 2d). Its prediction interval is 1–86%. The finding suggests that, on average, 35% of the adults in Latin America had insomnia symptoms during COVID-19 and the prevalence of insomnia symptoms in any comparable study will fall in this range.

The overall prevalence of mental health symptoms in frontline healthcare workers, general healthcare workers, the general population and students in Latin America was 37%, 34%, 33% and 45%, respectively. The overall prevalence rates of mental health symptoms that exceeded the cut-off values of mild, moderate and severe symptoms were 54%, 32% and 14%, respectively (Table 2). The pooled prevalence rates of mental health symptoms in South America, Central America, countries speaking Spanish and countries speaking Portuguese were 36%, 28%, 30% and 40%, respectively (Table 2). Subgroup analyses results on the anxiety, depression, distress and insomnia by population, severity, region and instrument are reported in Table 3.

**Table 2.** Pooled prevalence estimates of mental health symptoms by outcome, population, severity and region subgroups during the COVID-19 pandemic

First-level subgroup	Second-level subgroup	Prevalence (%)	95% CI (%)	*p* Value
Aggregated		35	32–37	<0.001
Outcome	Anxiety	35	31–38	<0.001
	Depression	35	31–39	<0.001
	Distress	32	25–40	<0.001
	Insomnia	35	25–46	<0.001
Population	Frontline HCWs	37	24–51	<0.001
	General HCWs	34	29–39	<0.001
	General population	33	30–37	<0.001
	Students	45	37–53	<0.001
Severity	Above mild	54	50–58	<0.001
	Above moderate	32	30–35	<0.001
	Above severe	14	12–17	<0.001
	Overall	32	22–44	<0.001
Region	South America	36	33–38	<0.001
	Central America	28	24–33	<0.001
	Countries speaking Spanish	30	27–33	<0.001
	Countries speaking Portuguese	40	36–43	<0.001
Quality	Studies with high quality	42	38–45	<0.001
	Studies with medium quality	31	28–34	<0.001

CI, confidence interval.

**Table 3.** Subgroup analyses of the prevalence of anxiety, depression and insomnia symptoms

Groups	Subgroups	Anxiety	Depression	Distress	Insomnia
Number of studies		54	46	13	7
Number of samples		56	49	13	9
Number of prevalence		95	87	21	19
Number of participants		128 060	139 559	10 335	12 134
Aggregated		35%, 95% CI: 31–39%	35%, 95% CI: 31–39%	32%, 95% CI: 25–40%	35%, 95% CI: 25–46%
Population	Frontline HCWs	23%, 95% CI: 21–26%	37%, 95% CI: 34–39%	NA	NA
	General HCWs	34%, 95% CI: 26–42%	34%, 95% CI: 25–44%	30%, 95% CI: 19–43%	34%, 95% CI: 21–47%
	General population	34%, 95% CI: 29–40%	33%, 95% CI: 28–37%	32%, 95% CI: 23–43%	NA
	Students	43%, 95% CI: 33–53%	54%, 95% CI: 42–65%	NA	31%, 95% CI: 2–75%
Severity	Above mild	55%, 95% CI: 48–61%	51%, 95% CI: 45–56%	55%, 95% CI: 42–67%	61%, 95% CI: 52–69%
	Above moderate	32%, 95% CI: 29–36%	32%, 95% CI: 28–37%	32%, 95% CI: 19–47%	32%, 95% CI: 27–37%
	Severe	14%, 95% CI: 11–17%	17%, 95% CI: 12–23%	17%, 95% CI: 13–22%	5%, 95% CI: 3–6%
Region	South America	37%, 95% CI: 32–41%	36%, 95% CI: 32–40%	33%, 95% CI: 25–41%	33%, 95% CI: 20–49%
	Central America	27%, 95% CI: 21–32%	27%, 95% CI: 20–34%	NA	45%, 95% CI: 37–53%
	Countries speaking Spanish	29%, 95% CI: 23–35%	29%, 95% CI: 25–34%	32%, 95% CI: 22–42%	34%, 95% CI: 21–48%
	Countries speaking Portuguese	40%, 95% CI: 35–45%	41%, 95% CI: 35–47%	33%, 95% CI: 21–46%	37%, 95% CI: 18–59%
Instrument		GAD: 32%, 95% CI: 26–38%	PHQ: 37%, 95% CI: 30–45%	CPDI: 35%, 95% CI: 10–65%	ISI: 32%, 95% CI: 17–49%
		DASS-21: 35%, 95% CI: 29–40%	DASS-21: 34%, 95% CI: 21–47%, *I*^2^: 99.9%	IES: 18%, 95% CI: 12–24%	DSM: 48%, 95% CI: 40–55%

CI, confidence interval.

### Quality of the studies

Of all studies, 30 studies (48.39%) were of high quality, and 32 studies (51.61%) were of medium quality (Table 1). The subgroup analysis suggests the high-quality studies reported a higher prevalence of mental health symptoms in Latin America (42%) than those of medium quality (31%) (Table 2).

### Detection of publication bias

**Fig. 3.**
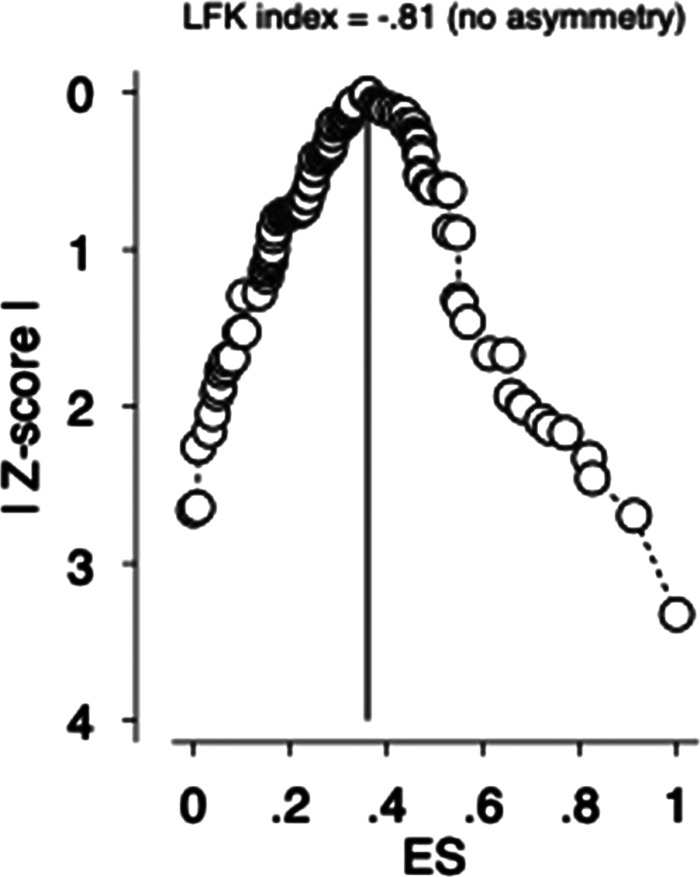
The Doi plot and the Luis Furuya–Kanamori (LFK) index for publication bias. ES, effect size.

The Doi plot and Luis Furuya–Kanamori index were used to quantify publication bias rather than the funnel plot and Egger's statistics (Furuya-Kanamori *et al*., 2018; Kounou *et al*., 2020). The symmetrical, hill-shaped Doi plot and a Luis Furuya–Kanamori (LFK) index of −0.81 indicated ‘no asymmetry’ and a lower likelihood of publication bias (Fig. 3).

## Discussion

The analysis of 62 studies with 196 950 participants from Latin America generated pooled prevalence of anxiety, depression, distress and insomnia of 35%, 35%, 32% and 35%, respectively. Notably, this meta-analysis is the first to investigate the prevalence of mental health symptoms during the COVID-19 crisis in Latin America. The anxiety levels in Latin America were significantly higher than other regions, such as China (25%; *p* < 0.001 (We compared the prevalence between two regions using *t*-test https://www.medcalc.org/calc/comparison_of_proportions.php)) (Ren *et al*., 2020) and Spain (20%; *p* < 0.001) (Chen *et al*., 2021*b*). Latin America has a long-standing history of scarce resources to deal with mental health symptoms (Alarcón, 2003), which could explain the higher prevalence of mental health symptoms among Latin Americans as revealed by this meta-analysis. Notably, the pooled prevalence of mental health symptoms was lower in Latin America than in Africa and South Asia, as reported by other meta-analyses (Hossain *et al*., 2020; Chen *et al*., 2021*a*). These cross-region differences may be due to multiple reasons, including heterogeneity in COVID-19 infection rate and mortality rate, variations in and timing of containment strategies adopted by countries across regions (Middelburg and Rosendaal, 2020), and the varying degrees of resources available, including personal protective equipment (PPE), to address mental health symptoms (Batra *et al*., 2020).

The prevalence of mental health symptoms was higher in South America than Central America (36% *v*. 28%; *p* < 0.001). This difference might be attributed to variations across these countries in the evolution of the pandemic (e.g. some countries such as Peru and Brazil started out well but deteriorated rapidly) (We appreciate a reviewer raising this point of discussion.), the provision and availability of PPE, healthcare facilities and capacities, the stringency of the COVID-19 responses and the political climate (Garcia *et al*., 2020). Previous research noted that South America generally has a high degree of political polarisation, which resulted in conflicting information being conveyed to the public that could increase the burden of COVID-19 and its associated psychological corollaries (Garcia *et al*., 2020). In addition, public health actions or decisions were made mostly at municipal and state levels rather than at central government levels, and the lack of central coordination posed several challenges in the control of the pandemic, contributing to an increased psychological burden (Garcia *et al*., 2020).

Based on the evidence of individual studies, our study found a higher prevalence of mental health symptoms among frontline HCWs (37%, *p* < 0.001) and university students (45%, *p* < 0.001) than the general population and general HCWs (Batra *et al*., 2020; Luo *et al*., 2020; Pappa *et al*., 2020). The vulnerabilities of frontline healthcare workers are often attributed to a higher risk of infection, burnout, the more direct exposure to suffering or dying patients, fear of COVID-19 transmission to their family members and job loss (Xiang *et al*., 2020; Bhandari *et al*., 2021). The greater prevalence of mental health symptoms among university students can be explained by the uncertainties surrounding the course of the pandemic and the sudden transition to online education (Adedoyin and Soykan, 2020; Batra *et al*., 2021). Moreover, many businesses scaled down their recruitment efforts, leading to limited employment opportunities for students and more competition in the graduate labour market (Reidy, 2020). These challenges added to the mental health burden among university students.

### Study limitations

There are a few limitations that merit discussion. First our analysis reveals substantial heterogeneities across studies in the type of survey instruments used and the cut-off scores, both of which may affect the interpretation of the findings. Second, not all Latin American countries have been well-studied, therefore our results may have limited generalisability for the less studied nations. Third, a majority of the included studies were cross-sectional, which provides no information on the prevalence over time during the pandemic. In addition, studies included in this meta-analysis relied on self-reported data of psychological symptoms by the participants and hence do not constitute mental health diagnosis from clinicians. Fourth, other outcomes, such as post traumatic stress disorder (PTSD), suicidal ideation and burnout, were not studied in this meta-analysis, leaving opportunities for prospective studies. Last, a language bias is expected because of the language restriction (only English) applied in this study. The systematic search uncovered eight papers (7.5%) that were not included for language reasons out of 107 eligible papers.

### Practical implications

First, our systematic review and meta-analysis support evidence-based medicine by revealing a high proportion of mental health symptoms among the general population and healthcare workers during the COVID-19 pandemic in Latin America. However, our systematic review also reveals there is a lack of evidence in many Latin American countries to guide the relevant practice of evidence-based medicine on this topic. Only 12 of the 33 Latin American countries have been studied, leaving 21 countries without any studies to assist the practice of evidence-based healthcare. For instance, no relevant research has been done in Venezuela, the fifth-biggest South American country with a population of 28 million, in Chile, the sixth biggest South American country with a population of 18 million, nor in Guatemala (18 million population), Cuba (11 million population) and the Dominican Republic (11 million population), respectively the second, fourth and fifth most populous countries in Central America. In practice, healthcare organisations in those unstudied countries may use our results in the same region as approximate evidence before direct evidence in those countries emerges.

Our findings that the prevalence of mental health symptoms was higher in South America than Central America (36% *v*. 28%; *p* < 0.001) provide evidence for international healthcare organisations, such as the World Psychiatric Association, on their assistance and resource allocation efforts. Our findings of a higher prevalence of mental health symptoms among frontline healthcare workers (37%, *p* < 0.001) and university students (45%, *p* < 0.001) than the general population (33%) and general healthcare workers (34%) suggest psychiatric and healthcare organisations should prioritise frontline healthcare workers and university students in Latin America.

## Conclusions

This meta-analysis, to our knowledge, provides the first pooled estimates of mental health symptoms among key demographic groups during the COVID-19 crisis in Latin America. The meta-analytical findings of this study underscore the high prevalence of mental health symptoms in Latin Americans during the COVID-19 crisis. Hence, we call for more research to identify people vulnerable to mental health symptoms to enable evidence-based medicine during the pandemic.

## Data Availability

The meta-analysis does not use primary data. All the secondary data that support the findings of this study are available from the corresponding author, J. C., upon request.

## Supplementary material

For supplementary material accompanying this paper visit https://doi.org/10.1017/S2045796021000767. click here to view supplementary material

## Patient and public involvement

No patient or public was involved in this systematic review and meta-analysis.

## References

[ref1] Adedoyin OB and Soykan E (2020) COVID-19 pandemic and online learning: the challenges and opportunities. Interactive Learning Environments 2020, 1–13.

[ref2] Alarcón RD (2003) Mental health and mental health care in Latin America. World Psychiatry 2, 54–56. 16946892 PMC1525063

[ref3] Antiporta DA, Cutipe YL, Mendoza M, Celentano DD, Stuart EA and Bruni A (2021) Depressive symptoms among Peruvian adult residents amidst a National Lockdown during the COVID-19 pandemic. BMC Psychiatry 21, 111–122. 33602157 10.1186/s12888-021-03107-3 PMC7890781

[ref4] Badellino H, Gobbo ME, Torres E and Aschieri ME (2020) Early indicators and risk factors associated with mental health problems during COVID-19 quarantine: is there a relationship with the number of confirmed cases and deaths? The International Journal of Social Psychiatry 67, 547–575. 10.1177/0020764020966020 PMC7565245 33054515

[ref5] Badellino H, Gobbo ME, Torres E, Aschieri ME, Biotti M, Alvarez V, Gigante C and Cachiarelli M (2022) ‘It's the economy, stupid’: lessons of a longitudinal study of depression in Argentina. The International Journal of Social Psychiatry 68, 1–8. 10.1177/0020764021999687 33706611

[ref6] Batra K, Singh TP, Sharma M, Batra R and Schvaneveldt N (2020) Investigating the psychological impact of COVID-19 among healthcare workers: a meta-analysis. International Journal of Environmental Research and Public Health 17, 9096. 10.3390/ijerph17239096 PMC7730003 33291511

[ref7] Batra K, Sharma M, Batra R, Singh TP and Schvaneveldt N (2021) Assessing the psychological impact of COVID-19 among college students: an evidence of 15 countries. Healthcare (Basel) 9, 222. 33671363 10.3390/healthcare9020222 PMC7923198

[ref8] Bhandari N, Batra K, Upadhyay S and Cochran C (2021) Impact of COVID-19 on healthcare labor market in the United States: lower paid workers experienced higher vulnerability and slower recovery. International Journal of Environmental Research and Public Health 18, 3894. 33917682 10.3390/ijerph18083894 PMC8067982

[ref9] Boluarte-Carbajal A, Navarro-Flores A and Villarreal-Zegarra D (2021) Explanatory model of perceived stress in the general population: a cross-sectional study in Peru during the COVID-19 context. Frontiers in Psychology 12, 673945. 34248770 10.3389/fpsyg.2021.673945 PMC8264254

[ref10] Borenstein M, Higgins JPT, Hedges LV and Rothstein HR (2017) Basics of meta-analysis: *I*^2^ is not an absolute measure of heterogeneity. Research Synthesis Methods 8, 5–18. 28058794 10.1002/jrsm.1230

[ref11] Borenstein LVH, Hedges LV, Higgins JPT and Rothstein HR (2021) Introduction to Meta-Analysis. Hoboken, NJ: John Wiley & Sons.

[ref12] Brito-Marques J, Franco CMR, de Brito-Marques PR, Martinez SCG and do Prado GF (2021) Impact of COVID-19 pandemic on the sleep quality of medical professionals in Brazil. Arquivos De Neuro-Psiquiatria 79, 149–155. 33759982 10.1590/0004-282X-anp-2020-0449

[ref13] Burki T (2020) COVID-19 in Latin America. The Lancet Infectious Diseases 5, 547–548. 10.1016/S1473-3099(20)30303-0 PMC7164892 32311323

[ref14] Campos JADB, Martins BG, Campos LA, Marôco J, Saadiq RA and Ruano R (2020) Early psychological impact of the COVID-19 pandemic in Brazil: a national survey. Journal of Clinical Medicine 9, 2976. 10.3390/jcm9092976 PMC7565796 32942647

[ref15] Campos J, Campos LA, Bueno JL and Martins BG (2021 *a*) Emotions and mood swings of pharmacy students in the context of the coronavirus disease of 2019 pandemic. Currents in Pharmacy Teaching and Learning 13, 635–642. 33867058 10.1016/j.cptl.2021.01.034 PMC7837624

[ref16] Campos J, Martins BG, Campos LA, de Fátima Valadão-Dias F and Marôco J (2021 *b*) Symptoms related to mental disorder in healthcare workers during the COVID-19 pandemic in Brazil. International Archives of Occupational and Environmental Health 94, 1023–1032. 33559748 10.1007/s00420-021-01656-4 PMC7871020

[ref17] Carvalho RA, Santos VS, Melo CM, Gurgel RQ and Oliveira CC (2015) Inequalities in health: living conditions and infant mortality in Northeastern Brazil. Revista de Saude Publica 49, 1–9. 25741650 10.1590/S0034-8910.2015049004794 PMC4386558

[ref18] Caycho-Rodriguez T, Tomas JM, Vilca LW, Garcia CH, Rojas-Jara C, White M and Pena-Calero BN (2022) Predictors of mental health during the COVID-19 pandemic in older adults: the role of socio-demographic variables and COVID-19 anxiety. Psychology Health & Medicine 27, 453–465. 10.1080/13548506.2021.1944655 34157907

[ref19] Cayo-Rojas CF, Castro-Mena MJ, Agramonte-Rosell RC, Aliaga-Mariñas AS, Ladera-Castañeda MI, Cervantes-Ganoza LA and Cervantes-Liñán LC (2021) Impact of COVID-19 mandatory social isolation on the development of anxiety in Peruvian dentistry students: a logistic regression analysis. Journal of International Society of Preventive & Community 11, 222–229. 10.4103/jispcd.JISPCD_52_21 PMC8118057 34036086

[ref20] Cénat JM, Dalexis RD, Guerrier M, Noorishad P-G, Derivois D, Bukaka J, Birangui J-P, Adansikou K, Clorméus LA and Kokou-Kpolou CK (2021) Frequency and correlates of anxiety symptoms during the COVID-19 pandemic in low-and middle-income countries: a multinational study. Journal of Psychiatric Research 132, 13–17. 33035760 10.1016/j.jpsychires.2020.09.031 PMC7527178

[ref21] Chen X, Zhang SX, Jahanshahi AA, Alvarez-Risco A, Dai H, Li J and Ibarra VG (2020) Belief in a COVID-19 conspiracy theory as a predictor of mental health and well-being of health care workers in Ecuador: cross-sectional survey study. JMIR Public Health Surveillance 6, e20737. 32658859 10.2196/20737 PMC7375774

[ref22] Chen J, Farah N, Dong RK, Chen RZ, Xu W, Yin A, Chen BZ, Delios A, Miller S, Wan X and Zhang SX (2021 *a*) The mental health under the COVID-19 crisis in Africa: a systematic review and meta-analysis. International Journal of Environmental Research and Public Health 18, 10604. 34682357 10.3390/ijerph182010604 PMC8536091

[ref23] Chen RZ, Zhang SX, Xu W, Yin A, Dong RK, Chen BZ, Delios A, McIntyre RS, Miller S and Wan X (2021 *b*) A systematic review and meta-analysis of symptoms of anxiety, depression, and insomnia in Spain in the COVID-19 crisis. International Journal of Environmental Research and Public Health 19, 1018. 10.3390/ijerph19021018 PMC8775436 35055841

[ref24] Civantos AM, Bertelli A, Gonçalves A, Getzen E, Chang C, Long Q and Rajasekaran K (2020) Mental health among head and neck surgeons in Brazil during the COVID-19 pandemic: a national study. American Journal of Otolaryngology 41, 102694. 32854041 10.1016/j.amjoto.2020.102694 PMC7442010

[ref25] Cortés-Álvarez NY, Pineiro-Lamas R and Vuelvas-Olmos CR (2020) Psychological effects and associated factors of COVID-19 in a Mexican sample. Journal Disaster Medicine Public Health Preparedness 14, 413–424. 32576317 10.1017/dmp.2020.215 PMC7385317

[ref26] Dal'Bosco EB, Floriano LSM, Skupien SV, Arcaro G, Martins AR and Anselmo ACC (2020) Mental health of nursing in coping with COVID-19 at a regional university hospital. Journal Revista Brasileira de Enfermagem 73, e20200434. 32667576 10.1590/0034-7167-2020-0434

[ref27] Dantas ESO, Araújo Filho JD, Silva G, Silveira MYM, Dantas MNP and Meira KC (2021) Factors associated with anxiety in multiprofessional health care residents during the COVID-19 pandemic. Revista Brasileira de Enfermagem 74S, e20200961. 33886846 10.1590/0034-7167-2020-0961

[ref28] da Silva Júnior AE, de Lima Macena M, de Oliveira ADS, Praxedes DRS, de Oliveira Maranhão Pureza IR and Bueno NB (2021) Racial differences in generalized anxiety disorder during the COVID-19 pandemic among Brazilian University Students: a national survey. Journal of Racial and Ethnic Health Disparities. doi: 10.1007/s40615-021-01107-3 PMC8294286 34291439

[ref29] Dávila-Cervantes CA and Agudelo-Botero M (2019) Health inequalities in Latin America: persistent gaps in life expectancy. The Lancet Planetary Health 3, 492–493. 10.1016/S2542-5196(19)30244-X 31836431

[ref30] De Boni RB, Balanzá-Martínez V, Mota JC, Cardoso TDA, Ballester P, Atienza-Carbonell B, Bastos FI and Kapczinski F (2020) Depression, anxiety, and lifestyle among essential workers: a web survey from Brazil and Spain during the COVID-19 pandemic. Journal of Medical Internet Research 22, e22835. 33038075 10.2196/22835 PMC7641648

[ref31] de Oliveira Andrade N, Correia Silva Azambuja H, Carvalho Reis Martins T, Manoel Seixas RA and Moretti Luchesi B (2021) Factors associated with depressive and anxiety symptoms in older adults during the COVID-19 pandemic: a Brazilian study. Aging & Mental Health 2021, 1–8. 10.1080/13607863.2021.1942431 34225507

[ref32] Espinosa-Guerra EA, Rodríguez-Barría ER, Donnelly CA and Carrera J-P (2021) Prevalence and associated factors with mental health outcomes among interns and residents physicians during COVID-19 epidemic in Panama: a cross-sectional study. medRxiv, 21254435.

[ref33] Esteves CS, de Oliveira CR and Argimon IID (2021) Social distancing: prevalence of depressive, anxiety, and stress symptoms among Brazilian students during the COVID-19 pandemic. Frontiers in Public Health 8, 5. 10.3389/fpubh.2020.589966 PMC7873553 33585381

[ref34] Fernández RS, Crivelli L, Guimet NM, Allegri RF and Pedreira ME (2020) Psychological distress associated with COVID-19 quarantine: latent profile analysis, outcome prediction and mediation analysis. Journal of Affective Disorders 277, 75–84. 32799107 10.1016/j.jad.2020.07.133 PMC7413121

[ref35] Fernandez MDS, Vieira IS, Silva N, Cardoso TA, Bielavski CH, Rakovski C and Silva AER (2021) Anxiety symptoms and alcohol abuse during the COVID-19 pandemic: a cross-sectional study with Brazilian dental undergraduate students. Journal of Dental Education 2021, 1–10. 10.1002/jdd.12742 PMC8426754 34268733

[ref36] Ferreira FD, Lopes-Silva JB, Siquara GM, Manfroi EC and de Freitas PM (2021) Coping in the COVID-19 pandemia: how different resources and strategies can be risk or protective factors to mental health in the Brazilian population. Health Psychology and Behavioral Medicine 9, 182–205. 34104556 10.1080/21642850.2021.1897595 PMC8158238

[ref37] Feter N, Caputo E, Doring I, Leite J, Cassuriaga J, Reichert F, da Silva M, Coombes J and Rombaldi A (2021) Sharp increase in depression and anxiety among Brazilian adults during the COVID-19 pandemic: findings from the PAMPA cohort. Public Health 190, 101–107. 33387848 10.1016/j.puhe.2020.11.013 PMC7773543

[ref38] Flores-Torres MH, Murchland AR, Espinosa-Tamez P, Jaen J, Brochier M, Bautista-Arredondo S, Lamadrid-Figueroa H, Lajous M and Koenen K (2021) Prevalence and correlates of mental health outcomes during the SARS-Cov-2 epidemic in Mexico City and their association with non-adherence to stay-at-home directives, June 2020. International Journal of Public Health 66, 1–10. 10.3389/ijph.2021.620825 PMC9847494 36688002

[ref39] Furuya-Kanamori L, Barendregt JJ and Doi SAR (2018) A new improved graphical and quantitative method for detecting bias in meta-analysis. International Journal of Evidence-Based Healthcare 16, 195–203. 29621038 10.1097/XEB.0000000000000141

[ref40] García-Espinosa P, Ortiz-Jiménez X, Botello-Hernández E, Aguayo-Samaniego R, Leija-Herrera J and Góngora-Rivera F (2021) Psychosocial impact on health-related and non-health related university students during the COVID-19 pandemic. Results of an electronic survey. Revista Colombiana de Psiquiatria 50, 214–224. 35400763 10.1016/j.rcp.2021.04.008 PMC8976581

[ref41] Garcia PJ, Alarcón A, Bayer A, Buss P, Guerra G, Ribeiro H, Rojas K, Saenz R, Snyder NSd, Solimano G, Torres R, Tobar S, Tuesca R, Vargas G and Atun R (2020) COVID-19 response in Latin America. The American Journal of Tropical Medicine and Hygiene 103, 1765–1772. 32940204 10.4269/ajtmh.20-0765 PMC7646820

[ref42] Giardino DL, Huck-Iriart C, Riddick M and Garay A (2020) The endless quarantine: the impact of the COVID-19 outbreak on healthcare workers after three months of mandatory social isolation in Argentina. Sleep Medicine 76, 16–25. 33059247 10.1016/j.sleep.2020.09.022 PMC7518855

[ref43] Goularte JF, Serafim SD, Colombo R, Hogg B, Caldieraro MA and Rosa AR (2021) COVID-19 and mental health in Brazil: psychiatric symptoms in the general population. Journal of Psychiatric Research 132, 32–37. 33038563 10.1016/j.jpsychires.2020.09.021 PMC7527181

[ref44] Guiroy A, Gagliardi M, Coombes N, Landriel F, Zanardi C, Camino Willhuber G, Guyot JP and Valacco M (2020) COVID-19 impact among spine surgeons in Latin America. Global Spine Journal 11, 859–865. 32875914 10.1177/2192568220928032 PMC8258821

[ref45] Higgins JPT, Thomas J, Chandler J, Cumpston M, Tianjing Li MJ and Page VAW (2019) Cochrane Handbook for Systematic Reviews of Interventions. Hoboken, NJ: John Wiley & Sons. 10.1002/14651858.ED000142 PMC10284251 31643080

[ref46] Hong QN, Fàbregues S, Bartlett G, Boardman F, Cargo M, Dagenais P, Gagnon M-P, Griffiths F, Nicolau B, O'Cathain A, Rousseau M-C, Vedel I and Pluye P (2018) The mixed methods appraisal tool (MMAT) version 2018 for information professionals and researchers. Education for Information 34, 285–291.

[ref47] Hossain M, Purohit N, Sultan A, Ma P, Lisako E, McKyer J and Ahmed HU (2020) Prevalence of mental disorders in South Asia: an umbrella review of systematic reviews and meta-analyses. Asian Journal of Psychiatry 51, 102041. 32315966 10.1016/j.ajp.2020.102041

[ref48] Kounou KB, Guédénon KM, Foli AAD and Gnassounou-Akpa E (2020) Mental health of medical professionals during the COVID-19 pandemic in Togo. Psychiatry and Clinical Neurosciences 74, 559–560. 32621390 10.1111/pcn.13108 PMC7361459

[ref49] Landaeta-Díaz L, González-Medina G and Agüero SD (2021) Anxiety, anhedonia and food consumption during the COVID-19 quarantine in Chile. Appetite 164, 105259. 33857546 10.1016/j.appet.2021.105259 PMC8050603

[ref50] Liberati A, Altman DG, Tetzlaff J, Mulrow C, Gøtzsche PC, Ioannidis JPA, Clarke M, Devereaux PJ, Kleijnen J and Moher D (2009) The PRISMA statement for reporting systematic reviews and meta-analyses of studies that evaluate healthcare interventions: explanation and elaboration. BMJ 62, e1–e34. 10.1136/bmj.b2700 PMC2714672 19622552

[ref51] Loret de Mola C, Blumenberg C, Martins RC, Martins-Silva T, Carpena MX, Del-Ponte B, Pearson R, Soares AL and Cesar JA (2021) Increased depression and anxiety during the COVID-19 pandemic in Brazilian mothers: a longitudinal study. Brazilian Journal of Psychiatry 43, 337–344. 33440402 10.1590/1516-4446-2020-1628 PMC8136383

[ref52] Luo M, Guo L, Yu M, Jiang W and Wang H (2020) The psychological and mental impact of coronavirus disease 2019 (COVID19) on medical staff and general public - a systematic review and meta-analysis. Psychiatry Research 291, 1–9. 10.1016/j.psychres.2020.113190 PMC7276119 32563745

[ref53] Malgor RD, Sobreira ML, Mouawad NJ, Johnson AP, Wohlauer MV, Coogan SM, Cuff RF, Coleman DM, Sheahan III MG and Woo K (2020) Brazilian vascular surgeons experience during the coronavirus (COVID-19) pandemic. Vascular 29, 451–460. 33019914 10.1177/1708538120954961 PMC7539231

[ref54] Malta M, Murray L, Da Silva CM and Strathdee SA (2020) Coronavirus in Brazil: the heavy weight of inequality and unsound leadership. EClinicalMedicine 25, 1–2. 10.1016/j.eclinm.2020.100472 PMC7380224 32754684

[ref55] Martinez EZ, Silva FM, Morigi TZ, Zucoloto ML, Silva TL, Joaquim AG, Dall'Agnol G, Galdino G, Martinez MOZ and Silva WRd (2020) Physical activity in periods of social distancing due to COVID-19: a cross-sectional survey. Ciência Saúde Coletiva 25, 4157–4168. 33027352 10.1590/1413-812320202510.2.27242020

[ref56] Mautong H, Gallardo-Rumbea JA, Alvarado-Villa GE, Fernández-Cadena JC, Andrade-Molina D, Orellana-Román CE and Cherrez-Ojeda I (2021) Assessment of depression, anxiety and stress levels in the Ecuadorian general population during social isolation due to the COVID-19 outbreak: a cross-sectional study. BMC Psychiatry 21, 1–15. 33910550 10.1186/s12888-021-03214-1 PMC8080088

[ref57] Medeiros RAD, Vieira DL, Silva EVFD, Rezende LVMDL, Santos RWD and Tabata LF (2020) Prevalence of symptoms of temporomandibular disorders, oral behaviors, anxiety, and depression in dentistry students during the period of social isolation due to COVID-19. Journal of Applied Oral Science 28, 1–8. 10.1590/1678-7757-2020-0445 PMC7714260 33263648

[ref58] Mendonca VS, Steil A and Gois AFT (2021) Mental health and the COVID-19 pandemic: a study of medical residency training over the years. Clinics 76, 1–6. 10.6061/clinics/2021/e2907 PMC8221564 34190854

[ref59] Middelburg RA and Rosendaal FR (2020) COVID-19: how to make between-country comparisons. International Journal of Infectious Diseases 96, 477–481. 32470605 10.1016/j.ijid.2020.05.066 PMC7250090

[ref60] Mier-Bolio JR, Arroyo-González JM, Baques-Guillén E, Valdez-Lopez JF, Torre-García ÁJ, Rodríguez-Rodríguez OE and Rivera-Arroyo G (2020) COVID-19 y ansiedad en oftalmólogos. Revista Mexicana de Oftalmologia 94, 223–227.

[ref61] Monterrosa-Castro A, Redondo-Mendoza V and Mercado-Lara M (2020) Psychosocial factors associated with symptoms of generalized anxiety disorder in general practitioners during the COVID-19 pandemic. Journal of Investigative Medicine 68, 1228–1234. 32747387 10.1136/jim-2020-001456

[ref62] Mora-Magaña I, Lee SA, Maldonado-Castellanos I, Jiménez-Gutierrez C, Mendez-Venegas J, Maya-Del-Moral A, Rosas-Munive MD, Mathis AA and Jobe MC (2020) Coronaphobia among healthcare professionals in Mexico: a psychometric analysis. Death Studies 1808762, 1–10. 10.1080/07481187.2020.1808762 32808877

[ref63] Mota IA, Oliveira Sobrinho GD, Morais IPS and Dantas TF (2021) Impact of COVID-19 on eating habits, physical activity and sleep in Brazilian healthcare professionals. Arquivos de Neuro-psiquiatria 79, 429–436. 34037163 10.1590/0004-282X-ANP-2020-0482 PMC9394556

[ref64] Nayak BS, Sahu PK, Ramsaroop K, Maharaj S, Mootoo W, Khan S and Extravour RM (2021) Prevalence and factors associated with depression, anxiety and stress among healthcare workers of Trinidad and Tobago during COVID-19 pandemic: a cross-sectional study. BMJ Open 11, e044397. 10.1136/bmjopen-2020-044397 PMC8050873 33849850

[ref65] Pablo GSd, Vaquerizo-Serrano J, Catalan A, Arango C, Moreno C, Ferre F, Shin JI, Sullivan S, Brondino N, Solmi M and Fusar-Poli P (2020) Impact of coronavirus syndromes on physical and mental health of health care workers: systematic review and meta-analysis. Journal of Affective Disorders 275, 48–57. 32658823 10.1016/j.jad.2020.06.022 PMC7314697

[ref66] Pappa S, Ntella V, Giannakas T, Giannakoulis VG, Papoutsi E and Katsaounou P (2020) Prevalence of depression, anxiety, and insomnia among healthcare workers during the COVID-19 pandemic: a systematic review and meta-analysis. Brain, Behavior, and Immunity 88, 901–907. 10.1016/j.bbi.2020.05.026 PMC7206431 32437915

[ref67] Passos L, Prazeres F, Teixeira A and Martins C (2020) Impact on mental health due to COVID-19 pandemic: cross-sectional study in Portugal and Brazil. International Journal of Environmental Research and Public Health 17, 1–13. 10.3390/ijerph17186794 PMC7557976 32957702

[ref68] Paz C, Mascialino G, Adana-Díaz L, Rodríguez-Lorenzana A, Simbaña-Rivera K, Gómez-Barreno L, Troya M, Páez MI, Cardenas J and Gerstner RM (2020) Anxiety and depression in patients with confirmed and suspected COVID-19 in Ecuador. Psychiatr Clinical Neurosciences 74, 554–555. 10.1111/pcn.13106 PMC7361296 32609409

[ref69] Puccinelli PJ, Costa TS, Seffrin A, de Lira CA, Vancini RL, Knechtle B, Nikolaidis PT and Andrade MS (2021) Physical activity levels and mental health during the COVID-19 pandemic: preliminary results of a comparative study between convenience samples from Brazil and Switzerland. Medicina 57, 48. 33429989 10.3390/medicina57010048 PMC7827202

[ref70] Reidy T (2020) Recruitment is on hold: the students graduating into the COVID-19 recession. *The Guardian*.

[ref71] Ren X, Huang W, Pan H, Huang T, Wang X and Ma Y (2020) Mental health during the COVID-19 outbreak in China: a meta-analysis. Psychiatric Quarterly 91, 1033–1045. 10.1007/s11126-020-09796-5 PMC7343383 32642822

[ref72] Ribeiro FS, Lessa JPA, Delmolin G and Santos FH (2021) Music listening in times of COVID-19 outbreak: a Brazilian study. Frontiers in Psychology 12, 10. 10.3389/fpsyg.2021.647473 PMC8177432 34093328

[ref73] Ríos AM (2021) Latin America: COVID-19 cases by country. Available at https://www.statista.com/statistics/1101643/latin-america-caribbean-coronavirus-cases/.

[ref74] Robles R, Rodríguez E, Vega-Ramírez H, Álvarez-Icaza D, Madrigal E, Durand S, Morales-Chainé S, Astudillo C, Real-Ramírez J and Medina-Mora M-E (2021) Mental health problems among healthcare workers involved with the COVID-19 outbreak. Brazilian Journal of Psychiatry 43, 494–503. 33331498 10.1590/1516-4446-2020-1346 PMC8555639

[ref75] Samaniego A, Urzúa A, Buenahora M and Vera-Villarroel P (2020) Sintomatología asociada a trastornos de salud mental en trabajadores sanitarios en Paraguay: efecto COVID-19. Revista Interamericana de Psicología/Interamerican Journal of Psycholog 54, e1298–e1298.

[ref76] Schmitt Jr AA, Brenner AM, Primo de Carvalho Alves L, Claudino FCdA, Fleck MPdA and Rocha NS (2021) Potential predictors of depressive symptoms during the initial stage of the COVID-19 outbreak among Brazilian adults. Journal of Affective Disorders 282, 1090–1095. 33601682 10.1016/j.jad.2020.12.203 PMC7832486

[ref77] Schuch FB, Bulzing RA, Meyer J, Vancampfort D, Firth J, Stubbs B, Grabovac I, Willeit P, Tavares VDO and Calegaro VC (2020) Associations of moderate to vigorous physical activity and sedentary behavior with depressive and anxiety symptoms in self-isolating people during the COVID-19 pandemic: a cross-sectional survey in Brazil. Psychiatry Research 292, 113339. 32745795 10.1016/j.psychres.2020.113339 PMC7384423

[ref78] Scotta AV, Cortez MV and Miranda AR (2021) Insomnia is associated with worry, cognitive avoidance and low academic engagement in Argentinian university students during the COVID-19 social isolation. Psychology Health Medicine 2021, 1–16. 10.1080/13548506.2020.1869796 33382639

[ref79] Serafim AP, Durães RS, Rocca CC, Gonçalves PD, Saffi F, Cappellozza A, Paulino M, Dumas-Diniz R, Brissos S and Brites R (2021) Exploratory study on the psychological impact of COVID-19 on the general Brazilian population. Plos One 16, e0245868. 33534820 10.1371/journal.pone.0245868 PMC7857630

[ref80] Souza ASR, Souza GFA, Souza GA, Cordeiro ALN, Praciano GAF, Alves ACS, Santos ACD, Silva Junior JR and Souza MBR (2021) Factors associated with stress, anxiety, and depression during social distancing in Brazil. Revista de Saude Publica 55, 5. 33852675 10.11606/s1518-8787.2021055003152 PMC8011840

[ref81] Torrente F, Yoris A, Low D, Lopez P, Bekinschtein P, Manes F and Cetkovich M (2021 *a*) Sooner than you think: a very early affective reaction to the COVID-19 pandemic and quarantine in Argentina. Journal of Affective Disorders 282, 495–503. 33422827 10.1016/j.jad.2020.12.124 PMC8529255

[ref82] Torrente F, Yoris A, Low D, Lopez P, Bekinschtein P, Vázquez G, Manes F and Cetkovich M (2021 *b*) Emotional symptoms, mental fatigue and behavioral adherence after 72 continuous days of strict lockdown during the COVID-19 pandemic in Argentina. medRxiv, 21255866. 10.1192/bjo.2021.1065 PMC8668400 34931146

[ref83] Usher K, Jackson D, Durkin J, Gyamfi N and Bhullar N (2020) Pandemic-related behaviours and psychological outcomes; a rapid literature review to explain COVID-19 behaviours. Journal of Mental Health Nursing 29, 1018–1034. 10.1111/inm.12790 32860475

[ref84] Villela EFD, da Cunha IR, Fodjo JNS, Obimpeh M, Colebunders R and Van Hees S (2021) Impact of COVID-19 on healthcare workers in Brazil between August and November 2020: a cross-sectional survey. International Journal of Environmental Research and Public Health 18, 6511–6522. 34204195 10.3390/ijerph18126511 PMC8296453

[ref85] Vitorino LM, Yoshinari GH, Gonzaga G, Dias IF, Pereira JPL, Ribeiro IMG, Franca AB, Al-Zaben F, Koenig HG and Trzesniak C (2021) Factors associated with mental health and quality of life during the COVID-19 pandemic in Brazil. BJPsych Open 7, 1–8. 10.1192/bjo.2021.62 PMC8129679 33988122

[ref86] Werneck AO, Silva DR, Malta DC, Souza-Júnior PR, Azevedo LO, Barros MB and Szwarcwald CL (2021) Physical inactivity and elevated TV-viewing reported changes during the COVID-19 pandemic are associated with mental health: a survey with 43,995 Brazilian adults. Journal of Psychosomatic Research 140, 110292. 33227555 10.1016/j.jpsychores.2020.110292 PMC7654295

[ref87] World Health Organization (2020) Global health estimates: Leading causes of death. Available at https://www.who.int/data/gho/data/themes/mortality-and-global-health-estimates/ghe-leading-causes-of-death.

[ref88] Xiang Y-T, Yang Y, Li W, Zhang L, Zhang Q, Cheung T and Ng CH (2020) Timely mental health care for the 2019 novel coronavirus outbreak is urgently needed. The Lancet. Psychiatry 7, 228–229. 32032543 10.1016/S2215-0366(20)30046-8 PMC7128153

[ref89] Yáñez JA, Jahanshahi AA, Alvarez-Risco A, Li J and Zhang SX (2020) Anxiety, distress, and turnover intention of healthcare workers in Peru by their distance to the epicenter during the COVID-19 crisis. The American Journal of Tropical Medicine and Hygiene 103, 1614–1620. 32815512 10.4269/ajtmh.20-0800 PMC7543861

[ref90] Yitayih Y, Mekonen S, Zeynudin A, Mengistie E and Ambelu A (2020) Mental health of healthcare professionals during the early stage of the COVID-19 pandemic in Ethiopia. The British Journal of Psychiatry 7, 1–6. 10.1192/bjo.2020.130 PMC7844150 33256883

[ref91] Zhang SX, Chen J, Jahanshahi AA, Alvarez-Risco A, Dai H, Li J and Patty-Tito RM (2021 *a*) Succumbing to the COVID-19 pandemic – healthcare workers not satisfied and intend to leave their jobs. International Journal of Environmental Research and Public Health 2021, 1–10.

[ref92] Zhang SX, Huang H, Li J, Antonelli-Ponti M, Paiva SFd and Silva JAd (2021 *b*) Predictors of depression and anxiety symptoms in Brazil during COVID-19. International Journal of Environmental Research and Public Health 18, 7026. 34209311 10.3390/ijerph18137026 PMC8297012

[ref93] Zhang SX, Wang Y, Jahanshahi AA, Li J and Schmitt VGH (2021 *c*) Early evidence and predictors of mental distress of adults one month in the COVID-19 epidemic in Brazil. Journal of Psychosomatic Research 142, 110366. 33494004 10.1016/j.jpsychores.2021.110366 PMC7816874

