# Supplementary material for: Mental disorder symptoms during the COVID-19 pandemic in Latin America – a systematic review and meta-analysis
Source: Epidemiol Psychiatr Sci. 2022 Apr 19;31:e23. doi: 10.1017/S2045796021000767 (PMC9069590; doi:10.1017/S2045796021000767)
Supplement: Supplementary file 1 [file S2045796021000767sup001.docx]

**Supplementary materials**

**Table S1. Search strategy for this systematic review and meta-analysis**

| Search | Search topic | Search keywords (titles, abstracts, and subject headings) with Boolean operators |
| --- | --- | --- |
| 1 | Exposure/ Context | 2019-nCoV OR 2019nCoV OR COVID-19 OR SARS-CoV-2 OR ((Wuhan AND coronavirus) |
| 2 | Outcome of interest | depressi* OR anxi* OR insomnia OR "sleep disorder" OR "sleep issue" OR "sleep problem" OR distress OR “mental health” OR psychiatric |
| 3 | Prevalence or empirical | “prevalen* OR "frequency" OR "ratio*" OR “proportion” |
| 4 | Countries or regions | brazil OR mexico OR colombia OR argentina OR peru OR venezuela OR chile OR guatemala OR ecuador OR bolivia OR haiti OR cuba OR “dominican republic” OR honduras OR paraguay OR nicaragua OR “el salvador” OR “costa rica” OR panama OR Uruguay OR jamaica OR “trinidad and Tobago” OR guyana OR suriname OR belize OR bahamas OR barbados OR “saint lucia” OR grenada OR “st. vincent & grenadines” OR “antigua and barbuda” OR dominica OR “saint kitts” OR “south america” OR “central America” OR “latin america” OR caribbean |
| 5 | Time | 2020.2.1 – 2021. 8.13 |
| Final search |  | 1 AND 2 AND 3 AND 4 AND 5 |

**Table S2. Studies included in this meta-analysis**

| Authors & Year | Country | Population | Sample size | Outcome | Instrument | Ethics statement |
| --- | --- | --- | --- | --- | --- | --- |
| Antiporta et al. 2021 | Peru | GP | 57446 | DEP | PHQ9 | Yes |
| Badellino et al. 2020 | Argentina | GP | 1985 | ANX, DEP | GAD7, PHQ9 | Yes |
| Badellino et al. 2021 | Argentina | GP | 1985 (t1),  2839 (t2) | DEP | PHQ9 | Yes |
| Boluarte-Carbajal et al. 2021 | Peru | GP | 204 | ANX, DEP, DIS | GAD7, PHQ9, PSS10 | Yes |
| Brito-Marques et al. 2021 | Brazil | GHCW | 332 | ANX, DEP, INS | GAD7, PHQ9, ISI | Yes |
| Campos et al. 2021a | Brazil | Student | 66 | ANX, DEP | DASS21 | Yes |
| Campos et al. 2020b | Brazil | GP | 12196 | ANX, DEP | DASS21 | Yes |
| Campos et al. 2021c | Brazil | GHCW | 1609 | ANX, DEP | DASS21 | Yes |
| Caycho-Rodriguez et al. 2021 | Peru | GP | 274 | ANX, DEP | GAD2, PHQ2 | Yes |
| Cayo-Rojas et al. 2021 | Peru | Student | 403 | ANX | The Zung Self-Rating Anxiety Scale | Yes |
| Cenat et al. 2020 | Haiti | GP | 225 | ANX | HSCL | Yes |
| Chen et al. 2020 | Ecuador | GHCW | 252 | ANX, DIS | GAD7, K6 | Yes |
| Civantos et al. 2020 | Brazil | GHCW | 163 | ANX, DEP, DIS | GAD7, PHQ2, IESR | Yes |
| Cortés-Álvarez et al. 2020 | Mexico | GP | 1105 | ANX, DEP, DIS | DASS21 | Yes |
| Dal’Bosco et al. 2020 | Brazil | FHCW | 88 | ANX, DEP | HADS | Yes |
| Da Silva Jãºnior et al. 2021 | Brazil | Student | 5879 | ANX | GAD7 | Yes |
| Dantas et al. 2021 | Brazil | GHCW | 67 | ANX | BAI | Yes |
| De Boni et al. 2020 | Brazil | GP | 2842 | ANX, DEP | GAD7, PHQ2 | Yes |
| De Oliveira Andrade et al. 2021 | Brazil | GP | 380 | ANX, DEP | GAI, GDS | Yes |
| Espinosa-Guerra et al. 2021 | Panama | GP | 512 | ANX, DEP, DIS | GAD7, PHQ9, IES-R | Yes |
| Esteves et al. 2021 | Brazil | Student | 208 | ANX, DEP, DIS | DASS21 | Yes |
| Fernández et al. 2020 | Argentina | GP | 4408 | ANX, DEP, DIS | BSI-53, GSI | Yes |
| Fernández et al. 2021 | Brazil | Student | 1050 | ANX | GAD-7 | Yes |
| Ferreira et al. 2021 | Brazil | GP | 1130 | ANX, DEP, DIS | DASS21 | Yes |
| Feter et al. 2021 | Brazil | GP | 1767 (t1), 2314 (t2) | ANX, DEP | HADS | Yes |
| García-Espinosa et al. 2021 | Mexico | Student | 1149 | ANX, DEP | GAD7, PHQ9 | Yes |
| Giardino et al. 2020 | Argentina | GHCW | 1059 | ANX, DEP, INS | GADS, ISI | Yes |
| Goularte et al. 2020 | Brazil | GP | 1996 | ANX, DEP, INS | DSM5 | Yes |
| Guiroy et al. 2020 | Mixed | GHCW | 204 | DEP | PHQ9 | No |
| Landaeta-Díaz et al. 2021 | Chile | GP | 1725 | ANX | BAI | Yes |
| Malgor et al. 2020 | Brazil | GHCW | 335 | ANX | GAD7 | Yes |
| Mario et al. 2021 | Mexico | GP | 2016 | ANX, DEP | GAD7, CES-D | Yes |
| Martinez EZ et al. 2020 | Brazil | GP | 1613 | ANX, DEP | HADS | Yes |
| Mautong et al. 2021 | Ecuador | GP | 626 | ANX, DEP, DIS | DASS21 | Yes |
| Medeiros et al. 2020 | Brazil | Student | 113 | ANX, DEP | HADS | Yes |
| Mendonca et al. 2021 | Brazil | Student | 3071 | ANX, DEP | GAD7, PHQ9 | Yes |
| Mier-Bolio et al. 2020 | Mexico | GHCW | 31 | ANX | GAD7 | Yes |
| Monterrosa-Castro et al. 2020 | Colombia | GHCW | 531 | ANX | GAD7 | Yes |
| Mora-Magana et al. 2020 | Mexico | GHCW | 231 | ANX, DEP | GAD2, PHQ4 | Yes |
| Mota et al. 2021 | Brazil | GHCW | 710 | INS | ISI7 | Yes |
| Nayak et al. 2021 | Trinidad & Tobago | GHCW | 395 | ANX, DEP | DASS21 | Yes |
| Passos et al. 2020 | Brazil | GP | 289 | ANX, DEP | GAD7, PHQ2 | Yes |
| Paz et al. 2020 | Ecuador | GP | 453 | ANX, DEP | GAD7, PHQ9 | Yes |
| Puccinelli et al. 2021 | Brazil | GP | 57 | ANX, DEP | GAD7, PHQ9 | Yes |
| Ribeiro et al. 2021 | Brazil | GP | 494 | ANX, DEP | GAD7, CES-D | Yes |
| Robles et al. 2020 | Mexico | FHCW, GHCW | 1389,  4420 | ANX, DEP, INS | ICD11 PHC, PHQ2, DSM | Yes |
| Samaniego et al. 2020 | Paraguay | GHCW | 126 | ANX, DEP | GAD7, PHQ9, ISIR | No |
| Schmitt et al. 2021 | Brazil | GP | 3274 | DEP | PHQ9 | Yes |
| Schuch et al. 2020 | Brazil | GP | 937 | ANX, DEP | BDI | Yes |
| Scotta & Miranda 2020 | Argentina | Student | 584 | INS | ISI | No |
| Serafim et al. 2020 | Brazil | GP | 3000 | ANX, DEP | DASS21 | Yes |
| Souza et al. 2021 | Brazil | GP | 3200 | ANX, DEP | DASS21 | Yes |
| Torrente et al. 2020 | Argentina | GP | 10053 | ANX, DEP | GAD7, PHQ9 | No |
| Torrente et al. 2021 | Argentina | GP | 3617 | ANX, DEP | GAD7, PHQ9 | Yes |
| Villela et al. 2021 | Brazil | GHCW | 295 | ANX, DEP | HADS-A, HADS-D | Yes |
| Vitorino et al. 2021 | Brazil | GP | 1156 | ANX, DEP | GAD7, PHQ9 | Yes |
| Werneck et al. 2020 | Brazil | GP | 43995 | ANX | NA | Yes |
| Yanez et al. 2020 | Peru | GHCW | 303 | ANX, DIS | GAD7, K6 | Yes |
| Zhang et al. 2020 | Peru | GP | 671 | DIS | CPDI | Yes |
| Zhang et al. 2021a | Brazil | GP | 482 | ANX, DEP | GAD7 | Yes |
| Zhang et al. 2021b | Brazil | GP | 638 | DIS | CPDI | Yes |
| Zhang et al. 2021c | Bolivia | GHCW | 240 | ANX, DEP | GAD7, K6 | Yes |

Note: GHCW = general healthcare workers, FHCW = Frontline healthcare workers, GP = general population, ANX = Anxiety, DEP= Depression, DIS = Distress, INS= Insomnia
